# Metabolic network alignment in large scale by network compression

**DOI:** 10.1186/1471-2105-13-S3-S2

**Published:** 2012-03-21

**Authors:** Ferhat Ay, Michael Dang, Tamer Kahveci

**Affiliations:** 1 Computer and Information Science and Engineering, University of Florida, Gainesville, FL 32611, USA; 2 Department of Genome Sciences, University of Washington, Seattle, WA 98195, USA

## Abstract

Metabolic network alignment is a system scale comparative analysis that discovers important similarities and differences across different metabolisms and organisms. Although the problem of aligning metabolic networks has been considered in the past, the computational complexity of the existing solutions has so far limited their use to moderately sized networks. In this paper, we address the problem of aligning two metabolic networks, particularly when both of them are too large to be dealt with using existing methods. We develop a generic framework that can significantly improve the scale of the networks that can be aligned in practical time. Our framework has three major phases, namely the *compression phase*, the *alignment phase *and the *refinement phase*. For the first phase, we develop an algorithm which transforms the given networks to a compressed domain where they are summarized using fewer nodes, termed *supernodes*, and interactions. In the second phase, we carry out the alignment in the compressed domain using an existing network alignment method as our base algorithm. This alignment results in supernode mappings in the compressed domain, each of which are smaller instances of network alignment problem. In the third phase, we solve each of the instances using the base alignment algorithm to refine the alignment results. We provide a user defined parameter to control the number of compression levels which generally determines the tradeoff between the quality of the alignment versus how fast the algorithm runs. Our experiments on the networks from KEGG pathway database demonstrate that the compression method we propose reduces the sizes of metabolic networks by almost half at each compression level which provides an expected speedup of more than an order of magnitude. We also observe that the alignments obtained by only one level of compression capture the original alignment results with high accuracy. Together, these suggest that our framework results in alignments that are comparable to existing algorithms and can do this with practical resource utilization for large scale networks that existing algorithms could not handle. As an example of our method's performance in practice, the alignment of organism-wide metabolic networks of human (1615 reactions) and mouse (1600 reactions) was performed under three minutes by only using a single level of compression.

## Background

Biological networks provide a compact representation of the roles of different biochemical entities and the interactions between them. Depending on the types of entities and interactions, these networks are segregated into different types, where each network type encompasses a particular set of biological processes. Protein-protein interaction (PPI) networks comprise binding relationships between two or more proteins to carry out specific cellular functions such as signal transduction. Regulatory networks consist of interactions between genes and gene products to control the rates at which genes are transcribed. Metabolic networks represent sets of chemical reactions that are catalyzed by enzymes to transform a set of metabolites into others to maintain the stability of a cell and to meet its particular needs. Analysis of the connectivity properties of these networks has proven to be crucial in uncovering the details of the cell machinery and in revealing the functional modules and complexes involved in this mechanism [1-4].

An essential type of network analysis is the comparative analysis that aims at identifying functionally similar elements or element sets shared among different organisms which would not be possible if these elements were only considered individually. This is often achieved through alignment of the networks of these organisms. Analogous to sequence alignment which identifies conserved sequences, network alignment reveals connectivity patterns that are conserved among two or more organisms. A number of studies have been done to systematically align different types of biological networks [5-21]. For metabolic networks, Pinter *et al*. [20] devised an algorithm that aligns query networks with specific topologies by using a graph theoretic approach. Recently, some of us developed an algorithm that combines both topological features and homological similarity of pairwise molecules to align metabolic networks [8]. We also proposed a method, SubMAP [9,10], that incorporates subnetwork mappings in metabolic network alignment. A similar method, IsoRank [21], has been applied to find the alignments of PPI networks. IsoRankN [11] extended this algorithm to work for multiple networks and to allow mappings of protein clusters.

Comparative analysis is important particulary for large metabolic networks such as organism-wide networks. Identification of the conserved patterns among metabolic networks across species provide insights for metabolic reconstruction of a newly sequenced genome [22], orthology detection [21], drug target identification [23] and identification of enzyme clusters and missing enzymes [24,25]. However, aligning large scale networks is a computationally challenging problem due to the underlying subgraph isomorphism problem that has to be solved to find the alignment that maximizes the similarity between the query networks. The methods we mentioned above either restrict the query topologies and/or their sizes. Even under these conditions, the running times and memory utilization of these methods can still be prohibitive for large query networks. For instance, the method of Pinter *et al*. [20] takes around one minute per alignment on a dataset with only small size networks ranging from 2 to 41 nodes. Our earlier method, SubMAP has no limitations on the query topologies and allows mappings of node sets that are connected (i.e., subnetworks). However, allowing subnetworks comes at a cost of increasing running time that is inherent due to the fact that the number of all connected subnetworks up to a given size can be exponential in the size of the network. For a network of size 80 and subnetwork sizes up to 3, SubMAP takes around 6 minutes and 150 MBs of memory on the average per alignment with a database of networks of size 50 on the average. Therefore, improving the running time and memory utilization of these methods is necessary to leverage the alignment of larger scale networks especially when subnetwork mappings are allowed.

**Figure 1:**
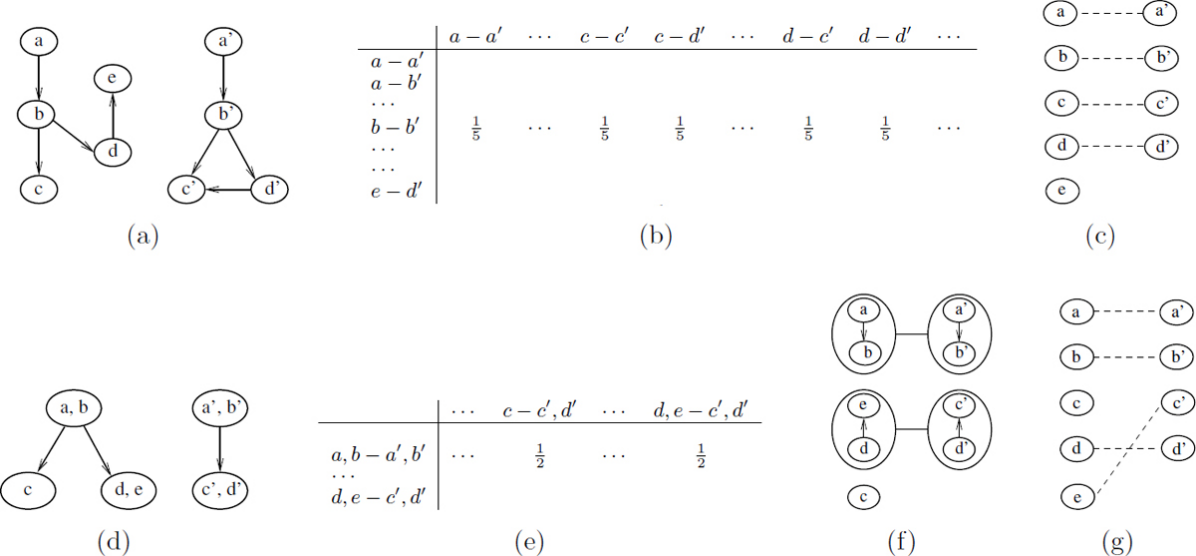
**Aligning two metabolic networks with and without compression.** Top figures (a-c) illustrate the steps of alignment without compression. Bottom figures (d-g) demonstrate different phases of alignment with compression using our framework. (a) Two hypothetical metabolic networks with 5 and 4 reactions respectively. Directed edges represent the neighborhood relations between the reactions. (b) Support matrix of size 20*×*20 needed for the alignment if compression is not used. We only show the non-zero entries of a single row that corresponds to topological support given by *b *- *b' *mapping to possible mappings of its backward and forward neighbors. Five such mappings supported equally are denoted by $\frac{1}{5}\text{s}$ in the matrix, namely *a *- *a' *mapping for the backward neighbors and *c *- *c'*, *c *- *d'*, *d *- *c' *and *d *- *d' *mappings for the forward neighbors. (c) The resulting reaction mappings of alignment without compression. (d) Query networks shown in (a) in compressed domain after one level of compression. (e) Support matrix of size 6*×*6 needed for the alignment with compression. We only show the entries for the mappings supported by the *a*, *b *- *a'*, *b' *mapping. (f) The resulting mappings from the alignment in compressed domain. (g) The resulting reaction mappings after refinement phase of our framework.

In this paper, we develop a framework that significantly improves the scale of the networks that can be aligned using existing algorithms. Our framework has three major phases, namely the *compression phase*, the *alignment phase *and the *refinement phase*. For the first phase, we develop a compression method that reduces the size of the input metabolic networks by a desired rate. In other words, we transform the query networks from their original domains (see Figure 1(a)) to a *compressed domain *(see Figure 1(d)). A single node in compressed domain corresponds to a set of connected nodes and the edges between them in the original domain. We call each such node in the compressed network a *supernode*. For instance, Figure 1(d) depicts the compressed networks of the two input networks in Figure 1(a) when each supernode is allowed to contain up to two nodes (i.e., only one level of compression is allowed). In the second phase, we carry out the alignment in the compressed domain by using an existing network alignment algorithm, which is SubMAP in this paper, as our base method. Once the compressed networks are aligned, we next consider each mapping of supernodes found by the first phase individually. Each such mapping suggests a smaller instance of network alignment. Figure 1(f) demonstrates this where two such instances exist. For each of these mappings, we solve the alignment problem using the base algorithm. At the end of this refinement phase, the final mappings of reactions are extracted (see Figure 1(g)) transforming the alignment back to the original domain.

We can best motivate the need for such a framework on an example. Figure 1 illustrates the difference between aligning two metabolic networks in compressed domain versus aligning them in the original domain without compression. If we use a base alignment algorithm such as SubMAP or IsoRank, the time and space complexity of the algorithm is determined by the size of a data structure, named *support matrix *[10,21]. Conceptually, this data structure governs the topological similarities between every pair of reaction tuples. Each reaction tuple contains one reaction from each of the two query metabolic networks. A detailed description of this matrix can be found in previous articles describing IsoRank [21] and SubMAP [10]. The size of this support matrix is quadratic in terms of both *n *and *m *(i.e., *O *(*n*^2^*m*^2^)) for IsoRank and for SubMAP when only subnetworks of size one are allowed. Figures 1(b) and 1(e) illustrate the support matrices required for alignment starting from the networks shown in Figure 1(a) and 1(d) respectively. As a result of compression by only one level, the size of the matrix we need to create, drops to 6*×*6 from 20*×*20 which translates into more than an order of magnitude improvement in theoretical resource utilization compared to the base method.

Notice that when we compress the network more (i.e., increase the number of compression levels), the compressed network gets smaller in terms of its number of nodes and edges. As a result, we can expect to align the compressed networks faster. However, this comes at the price of two drawbacks both due to the fact that each supernode contains multiple nodes from the original domain. First, once we find a mapping for the supernodes in the compressed domain, we still need to align the nodes of each supernode pair. For example, after mapping the supernodes (a, b) and (*a'*, *b'*) shown in Figure 1(f), we need to align the two subnetworks induced by these two supernodes. Thus as the size of the supernodes grow (i.e., as we compress for more levels), the size of the smaller problem instances grow as well and resource utilization bottleneck shifts from the alignment phase to refinement phase. Second, when we use compression the resulting alignment may not be the same as the one found by the original algorithm. For example, one out of four mappings in Figure 1(g) (i.e., *e *- *c'*) is different than the results of the base algorithm shown in Figure 1(c) (i.e., *e *- *e'*). This brings the need to define a measure of consistency between the results of alignments with and without compression which can be used as an indicator of accuracy for the framework we propose here. We calculate this accuracy as the correlation of the scores calculated for each possible mapping found by our framework in the compressed domain with the scores for these mapping in the original domain found by the base method. Bigger compression rates generally mean less similarity between the results of the two methods (i.e., less accuracy).

Several key questions follow from these observations are:

1. How does compression affect the alignment accuracy with respect to the base network alignment method?

2. How far is our compression method from an optimal compression that produces the compressed network with the minimum number of nodes?

3. When is it a good idea to do the alignment in compressed domain taking into account the overhead of compression and refinement phases?

4. What is the right amount of compression? That is, when does compression minimize the running time of our overall framework?

In the rest of the paper we address each of these questions in detail. At this point, it is important to notice the potential for leveraging the alignment of larger scale networks by the framework we are proposing. The actual performance gain for an alignment will depend on the level of compression we use, the topologies of the query networks and complexity of the base alignment method.

### Results overview

Our experiments on metabolic networks extracted from KEGG pathway database [26] demonstrate that our compression method reduces the number of nodes and edges by almost half at each level of compression. As a result of this reduction, we observe significant amount of improvement in running time and memory utilization of our earlier alignment algorithm SubMAP. Lastly, we analyze the accuracy of our framework as compared to the base alignment algorithm. The results suggest that the alignment obtained by only one level of compression captures the original alignment results with very high accuracy and the accuracy decreases with further levels of compression.

### Technical contributions

- We devise an efficient framework for the network alignment problem that employs a scalable compression method which shrinks the given networks while respecting their topology.

- We prove the optimality of our compression method under certain conditions and provide a bound on how much our compression results can deviate from the optimal solution in the worst case.

- We provide a mathematical formulation that serves as a guideline to select an optimal number of compression levels depending on the input characteristics of the alignment.

- We characterize the cases for which the proposed framework is expected to provide significant improvement in alignment performance.

In the next section, we report our experimental results on a set of large scale metabolic networks that are constructed by combining networks from KEGG Pathway database [26]. The details of the network compression method we propose here and the other phases of our framework are described in the methods section.

## Results and discussion

In this section, we experimentally evaluate the performance of our framework. First, we measure the compression rates achieved for different levels of compression with minimum degree selection (*MDS*) method that we propose here.

Next, we further analyzed the changes in degree distribution and large scale organization of organism-wide metabolic networks with increasing compression levels. We, then, examine the gain in running time and memory utilization achieved by our framework for different values of compression level (*c*) and subnetwork size (*k*) parameters. Last, we examine the accuracy of the alignments we found by measuring the accuracy as the Pearson's correlation coefficient between the scores of mappings calculated by our framework and the ones calculated by the base algorithm we use.

### Dataset

We use the metabolic networks from the KEGG pathway database [26]. For our *medium scale dataset*, we downloaded all metabolic networks with at least 10 reactions for 10 different organisms. This resulted in 620 metabolic networks in total with sizes ranging from 10 to 97.

In order to obtain our *large scale dataset*, we first combined all the metabolic networks that belong to one of the 9 different metabolism categories in KEGG database to create a *complete metabolism network *for each metabolism for 10 selected organisms (Homo sapiens (human), Mus musculus (mouse), Rattus norvegicus (rat), Drosophila melanogaster (fruit fly), Arabidopsis thaliana (thale cress), Caenorhabditis elegans (nematode), Saccharomyces cerevisiae (budding yeast), Staphylococcus aureus COL (MRSA), Escherichia coli K-12 MG1655, Pseudomonas aeruginosa PAO1). We obtain the *organism-wide metabolic networks *by combining all the listed networks in KEGG for each of these organisms. In total, we have 100 networks with sizes ranging from 5 to 1615 (9 complete metabolism networks plus 1 organism-wide network for each of the 10 organisms). Below is the list of metabolism categories we use.

1. Carbohydrate Metabolism

2. Energy Metabolism

3. Lipid Metabolism

4. Nucleotide Metabolism

5. Amino Acid Metabolism

6. Metabolism of Other Amino Acids

7. Glycan Biosynthesis and Metabolism

8. Metabolism of Cofactors and Vitamins

9. All Amino Acids (Amino Acid + Other Amino Acids)

### Implementation and system details

We implemented our compression and alignment algorithms in C_++_. We ran all the experiments on a desktop computer running Red Hat Enterprise Client 5.7 with 4 GB of RAM and two dual-core 2.40 GHz processors.

#### Evaluation of compression rates

The efficiency of our alignment framework depends on how much the query metabolic networks can be compressed. For this reason, in this experiment, we measure the number of nodes and edges of the metabolic networks in our large scale dataset before and after compression.

The minimum degree selection (*MDS*) method we describe in this paper compresses the query metabolic networks by selecting the first node among the list of nodes with minimum degree at each intermediate step and by compressing it with one of its neighbors. In order to evaluate stability of this compression method, we examined the effect of the node selection strategy on the size of the resulting compressed networks. By randomizing the step at which we select a node among the set of minimum degree nodes, we generated 100 different compressed networks for each of the input metabolic networks. In the following, we examine how much compression we achieve by the *MDS *method and also analyze its stability with respect to compressions achieved by randomization of node selection step.

Table 1 summarizes the compression rates achieved by our method for networks of different sizes. We divide all the metabolic networks in our dataset into bins according to the number of their reactions (i.e., network size). The first column in Table 1 lists the network size intervals we used for each group. Notice that the gaps in the size interval are due to the fact that organism-wide networks are of size 850 and larger whereas the other combined networks for nine different metabolism categories have sizes below 400. Each row of this table shows the number of nodes and edges averaged over all the networks in this group before and after compression. The two columns with *c *= 0 correspond to the average number of nodes and edges of the networks with no compression respectively. For *c *∈ {1, 2, 3}, we split each row corresponding to an interval into two. The upper part denotes the average node and edge numbers for the compressed network if the *MDS *method is used as originally described (i.e., the first among the list of minimum degree nodes is selected and combined with its first neighbor at each compression step). The lower part in bold represents the numbers gathered when we introduce randomization in this node selection. Each value in bold in Table 1 denotes the average of the corresponding value over these 100 different runs of compression.

**Table 1:** Summary of compression rates for all the networks in our large scale dataset

*Network size intervals*	*Average number of nodes*				*Average number of edges*			
	**c = 0**	**c = 1**	**c = 2**	**c = 3**	**c = 0**	**c = 1**	**c = 2**	**c = 3**

[0, 100)	41.5	26.5 **26.5**	19.1 **19.1**	15 **14.8**	83.5	55.2 **55.5**	36.3 **36.5**	23.6 **23.5**
[100, 200)	154.8	92.4 **92.2**	61.3 **61.5**	48.6 **48.6**	310.1	174.9 **174**	116.5 **118.1**	96.3 **94.6**
[200, 300)	240.5	139.1 **139.4**	89.2 **89.1**	69.4 **69.7**	508.1	296.5 **298.4**	230.5 **228.4**	187.8 **188.1**
[300, 400]	344.9	207.3 **207.6**	133.1 **133.8**	103 **104.5**	585.7	372.9 **373.5**	302.7 **300.4**	261.6 **259.9**
[850, 1250]	1080.5	623.2 **623.7**	406.8 **407.9**	311.3 **311.9**	3727	2269 **2280.6**	1732.7 **1733.8**	1584.8 **1587.5**
[1500, 1615]	1576.5	909 **910**	582 **583**	447.8 **444.6**	4740	2955.2 **2964.3**	2283.5 **2279.3**	2128.8 **2129.6**

We create six intervals according to number of reactions in these networks. Each row, corresponding to one such interval, shows the average number of nodes and edges before compression (i.e., *c *= 0) and after compression of different levels (i.e., *c *∈ {1, 2, 3}). For each row, top entries correspond to numbers obtained with the *M D S *method which selects the first node from the list of nodes with minimum degree at each intermediate step and compresses it with its first neighbor from the list of its neighbors. The bottom entries that are in **bold **correspond to the averages of 100 different compressions which are gathered by randomizing the step at which a node is selected among the set of minimum degree nodes.

One conclusion that can be drawn from Table 1 is that independent of the network size, our compression method performs well in practice. On the average, with only one level of compression we achieve network sizes that are 57-64%, 64-71% and 77-80% of the network sizes in the previous compression level for *c *= 1, 2 and 3 respectively. In other words, our method compresses the entire dataset down to approximately 60%, 40% and 30% of the sizes of original networks for *c *= 1, 2 and 3 respectively. These rates suggest that our framework has great potential in scaling the network alignment to large metabolic networks by compression. As an example, consider the row corresponding to interval [850,1250] in Table 1. We see that instead of aligning networks with 1080 nodes and 3727 edges on the average, we can apply two levels of compression first and do the alignment with significantly smaller networks that have only 407 nodes and 1733 edges on the average. Another observation is that, we get the most of the reduction in network size after the first compression level. That is, our method compresses the networks aggressively for *c *= 1 and achieves 57% to 64% compression rate which is close to the half of the size of the networks. As we go up in the levels of compression, the actual rate of compression achieved at one level reduces. Considering the fact that having an input network which can lead to the best possible compression (i.e., reducing its size from *n *down to size $\left\lceil \frac{n}{2}\right\rceil$ (i.e., 50%) at each level of compression) is a rare event, the observed compression rates suggest that our method provides an efficient compression for metabolic networks in practice.

This experimental setup also suggests that the *MDS *method is stable with respect to the choice of the node to compress as long as that node is selected among the nodes with minimum degree. Among the six rows and three columns (18 entries) of Table 1 for the average number of nodes after the compression, only one of them have difference larger than two between the original size and the randomized average.

*The results of this experiment suggest that our compression method, MDS, serves as an efficient and stable first phase for our alignment framework by achieving good compression rates on a large dataset of metabolic networks*.

#### Changes in degree distributions with compression

Even though the compression rates we achieve with *MDS *as described above suggest significant reduction in the problem size, we observe that there is a noticeable difference between the compression rates achieved by going from one compression level to the next. For instance, on the average we get 57% to 64% reduction in the size of the networks going from *c *= 0 to *c *= 1 whereas we only get 76% to 80% reduction if we go from *c *= 2 to *c *= 3. This suggests that the large scale organization of the networks change with increasing levels of compression. Even though a change in the network structure can be expected as a result of our compression, it is not obvious how to quantify this change and whether the change is consistent among different metabolic networks.

**Figure 2:**
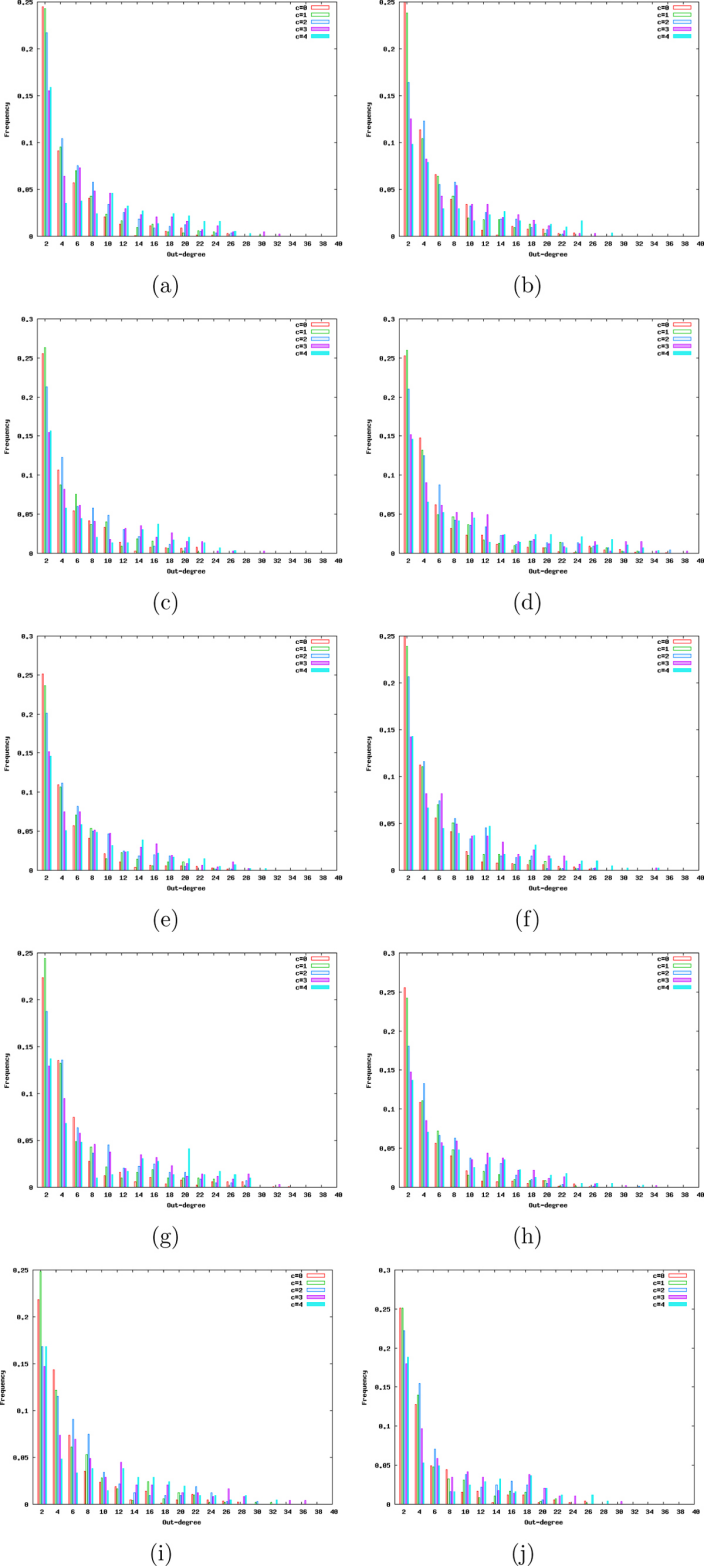
**Shift of out-degree distributions from power law to uniform.** Changes in the out-degree distributions of ten organism-wide metabolic networks with increasing levels of compression. We calculate the frequencies of each out-degree in the range [2,40] for *c *∈ {0, 1, 2, 3, 4} and plot them together for each of the ten organisms in our dataset. Out-degree distributions for organism-wide metabolic networks of (a) *Arabidopsis thaliana *(thale cress), (b) *Caenorhabditis elegans *(nematode), (c) *Drosophila melanogaster *(fruit fly), (d) *Escherichia coli K-12 MG1655*, (e) *Homo sapiens *(human), (f) *Mus musculus *(mouse), (g) *Pseudomonas aeruginosa PAO1*, (h) *Rattus norvegicus *(rat), (i) *Staphylococcus aureus COL *(MRSA), (j) *Saccharomyces cerevisiae *(budding yeast).

In order to understand the reason behind different compression rates for different compression levels, we examined the degree distributions of the ten organism-wide networks we have in our dataset. For each of these networks, we plotted the histogram of out-degree distributions for different levels of compression. Figure 2 plots the frequencies of each out-degree in the range [2,40] for each *c *∈ {0, 1, 2, 3, 4} for these networks. We observe that for each of these plots the degree distributions for *c *= 0 and *c *= 1 are very similar and they follow power-law distribution which is an indicator of scale-free network topology. This is not surprising since the scale-free topology has been observed in numerous articles in the literature as a common signature for different metabolic networks [27-29]. The similarity between the degree distributions of the original networks (*c *= 0) and the networks compressed by only one level (*c *= 1) signifies that the networks still conserve their scale-freeness after the first level of compression.

A more interesting observation is that there is a consistent shift from the power-law degree distribution to uniform distribution with increasing *c *values for each of the ten networks we have. It is important to clarify that our claim is not that the degree distribution becomes uniform for large *c *values but rather the degree distributions for large *c *values are more similar to uniform distribution (also less similar to power-law distribution) compared to ones obtained with smaller *c *values. To quantify this on an example, we look at one of the most discernable characteristics of scale-free networks, hence the power-law distribution, which is the small number of hub nodes with large degrees. If we consider the organism-wide network of *Homo sapiens *(Figure 2(e)), which is the largest network in our dataset, and focus on the percentage of nodes with out-degree greater than 15, we get percentages of 3%, 4%, 6.5%, 11.5% and 12.4% for *c *values of 0, 1, 2, 3 and 4 respectively. This indicates that the number of nodes that can be considered as hubs increase significantly with increasing levels of compression. This increase deteriorates the scale-freeness of the *Homo sapiens *network which in turn decreases the achieved compression rates. Similar trend is observed for each of the other nine organism-wide networks which are plotted separately in Figure 2.

*The results of this experiment show that there is a consistent change in the network topology when multiple levels of compression is used. This difference we observe here between the first level of compression and later levels of compression is likely to be one of the main reasons of the significant differences in both the performance and the accuracy of our framework which will be discussed next in the remaining of the results section*.

#### Evaluation of running time and memory utilization

In order to understand the capabilities and limitations of our framework, we examine its performance in terms of its running time and memory utilization on a set of large scale networks we constructed as described in the dataset section. We have ten networks for each of the ten organisms in our dataset. For each organism, nine of these networks constitute different metabolism categories and the tenth network is the organism-wide metabolic network. In total, we have 100 networks with sizes ranging from 5 to 1615. For each parameter setting (different combinations of *k *∈ {1, 2} and *c *∈ {0, 1, 2, 3}, we aligned each of these 100 networks with each other network (including itself) resulting in a total of 5500 alignment queries. When the value of *c *is equal to zero, the alignment is carried out completely by a single application of SubMAP without any compression. This provides us a mechanism to measure how much performance gain is achieved by our compression based framework with respect to SubMAP.

**Figure 3:**
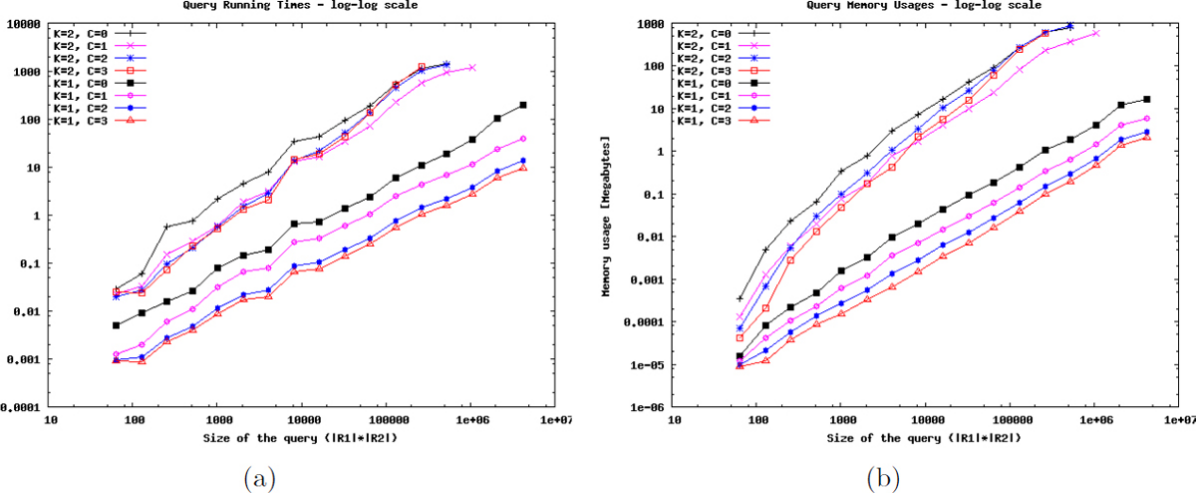
**Resource utilization of our framework.** The average (a) running time and (b) memory utilization of our framework when each query network in our large scale dataset is aligned with all the networks (including itself) in the same dataset. x-axis is the query size which is calculated as the product of the sizes (i.e., number of reactions) of the metabolic networks aligned. *c *= 0 denote the alignments performed with no compression. *c *∈ {1, 2, 3} denote the results of our framework that compresses both of the query networks by *c *levels before aligning them.

Figure 3(a) illustrates the average query running times in a log-log plot where x-axis is the size of the query measured as the product of the number of reactions of the metabolic networks that are aligned. We grouped queries into logarithmic bins according to the query sizes. The first bin contains all the queries of size less than or equal to 64. The next bins contain the queries of size in the interval [2^*i*+5^, 2^*i*+6^] where *i *= 2, 3, ..., 17. For each parameter setting we display the average running time of all the queries in each bin. For both *k *= 1 and *k *= 2, we plot all the results for all four different compression values and also draw the fitting curves to better illustrate the trend in the increase of running time.

For *k *= 1, we can immediately observe that each additional compression level improves the running time over the previous one for all query sizes. We obtain the largest fold change in running time by only one level of compression for the first level. This is expected considering that the first level of compression achieved the largest compression rate as shown in Table 1. The second compression level improves the running time by a smaller factor compared to the first and by a larger factor compared to the third level. For *k *= 1 we were able to plot all the points for all *c *values as the running time for even the largest query (i.e., human organism-wide network vs itself which has size 1615*1615) with no-compression (i.e., *c *= 0) is still practical, around 12 minutes (with *c *= 3 this drops to *<*40 seconds).

**Figure 4:**
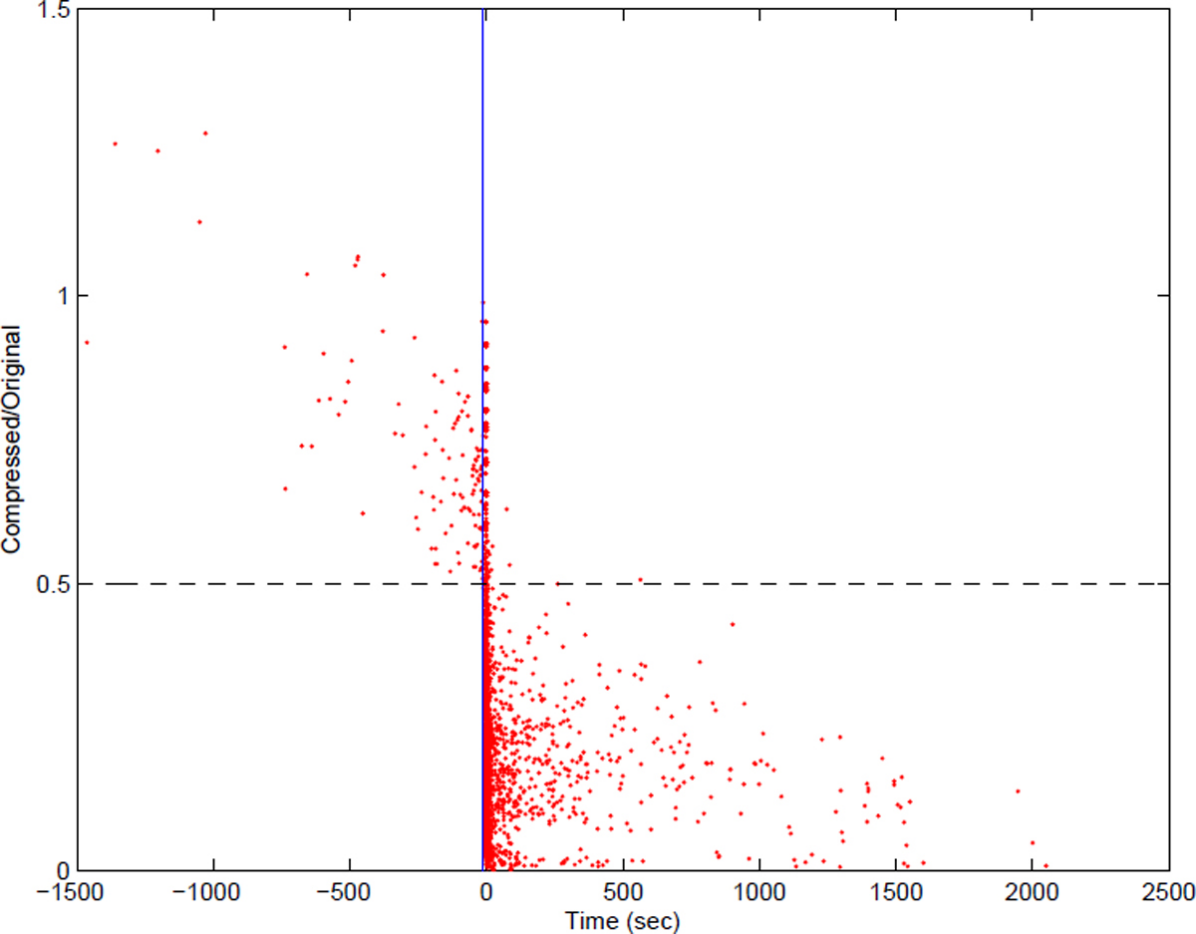
**Gain/Loss in running time.** Gain/Loss in running time of alignment by using our framework with respect to the base alignment method (x-axis) versus the ratio of the number of all possible subnetwork mappings in compressed domain to this number in the original domain. The blue vertical line shows when the two methods take exact same amount of time or when both methods take very short amount of time in the case of small query networks. Points on the right (left) handside of this line means gain (loss) in the running time. The dashed line is our decision criteria for predicting whether there will be gain or loss before doing the alignment.

Similar trend of improved running times with increasing *c *is also observed for queries up to a certain size for *k *= 2. For only one level of compression (*c *= 1) we observe significant improvement in running times for queries of all different sizes. However, starting from the bin [2^13^, 2^14^] compressing the networks more than only one level (*c >*1) shows a consistent adverse effect on the running time. This implies when both query networks have sizes around 150 or larger and *k >*1 is used, the idea of compressing the networks more than one level and then performing the alignment suffers from the explosion in the number of possible subnetworks in the compressed domain with size at most *k*. We explore this in more detail later on in the paper (see Figure 4 and its discussion).

An important aspect of our framework is that it makes possible to align networks that could not be aligned with our base method. For *k *= 2, we observed that in the original domain (*c *= 0) a significant portion of the large queries did not finish in less than the cutoff time which we set as one hour. For instance, among 252 possible queries with sizes in the interval [2^17^, 2^18^], 96 did not complete successfully for *c *= 0 whereas with *c *= 1 all of them were completed. For the next bin, 45 out of 223 possible queries were completed for *c *= 0 and for *c *= 1 this number increased to 185. These results indicate that by using the correct amount of compression, we can align larger networks than the base alignment method SubMAP. We believe this is an important step in leveraging organism-wide network alignments with subnetwork mappings for they provide a more complete picture of functional similarities and evolutionary differences between the metabolic networks of two or more organisms.

Figure 3(b) presents results for the estimated memory required for the support matrix, which is the memory bottleneck of the algorithm, that is needed to perform the alignment. For this figure, we use the same query set as Figure 3(a), hence the same x-axis. On the average the memory required for alignment with *c *= 1 is around 30% of that needed for alignment with no compression using the SubMAP method for both *k *= 1 and *k *= 2. For *k *= 1, the memory utilization decreases by each additional compression level (on the average around 45% of the memory required for *c *= 1 is used when *c *is increased to 2 and around 65% of the memory required for *c *= 2 is used when *c *is increased to 3). For *k *= 2, concordance with the running time results, only one level of compression provides better memory utilization for all network sizes whereas compressing more than one level has an adverse effect for medium and large scale queries.

*These results suggest that our framework demonstrates a great potential to provide significant improvement in both the running time and the memory utilization of the base alignment method. This allows us to align large networks that could not be aligned by existing methods by utilizing the same hardware*.

#### Accuracy of the alignment results

We conclude our experimental results by answering the first question introduced earlier in the paper, that is "How does compression affect the alignment accuracy?". In order to answer this, we calculate the correlation between the scores of each possible mapping in compressed domain and the scores that we obtain for these mappings from the original SubMAP method. We consider the scores of each possible subnetwork mapping of compressed nodes found by our framework. Since the mappings found by SubMAP are not of the same form with the mappings in compressed domain, we calculate a score value for each mapping in compressed domain by using the scores of the mappings found by SubMAP in the original domain. This way, we get two sets of score values one from SubMAP one from our framework for the same set of mappings. We calculate the Pearson's correlation coefficient between these two sets of scores as an indicator of the similarity between the results of the two methods.

Before looking at the correlation values we found, it is important to describe how we calculate the score for a mapping in compressed domain from the mappings of SubMAP. Let *P*^1 ^and ${\bar{P}}^{1}$ denote the one level compressed forms of two metabolic networks. Let $\left(v_{1^{-}}\left\{{\bar{v}}_{1},{\bar{v}}_{2}\right\}\right)$ denote a mapping in compressed domain where *v*_1 _is a subnetwork of *P*_1 _and $\left\{{\bar{v}}_{1},{\bar{v}}_{2}\right\}$ is a subnetwork of ${\bar{P}}^{1}$. Also, let *v*_1 _= {*r*_1_, *r*_2_}, ${\bar{v}}_{1}=\left\{{\bar{r}}_{1},{\bar{r}}_{2}\right\}$ and ${\bar{v}}_{2}=\left\{{\bar{r}}_{3}\right\}$. We know the edge that maps these two subnetworks has a mapping score in the compressed domain and let us denote it by |*e*^1^} for *c *= 1. We want to compute a mapping score, say |*e*|, for $\left(v_{1}\;\text{-}\;\text{\{}{\bar{v}}_{1},{\bar{v}}_{2}\}\right)$ from the mappings in original domain that is comparable to |*e*^1^|. This subnetwork mapping in compressed domain contains six possible mappings in the original, namely $\left(r_{1},\;{\bar{r}}_{1}\right)$, $\left(r_{1},\;{\bar{r}}_{2}\right)$, $\left(r_{1},\;{\bar{r}}_{3}\right)$, $\left(r_{2},\;{\bar{r}}_{1}\right)$, $\left(r_{2},\;{\bar{r}}_{2}\right)$ and $\left(r_{2},\;{\bar{r}}_{3}\right)$. Let us denote the scores of these mappings in the original domain by |*e_i_*| for *i *= 1, 2, ..., 6 respective to their ordering. Then, we compute the mapping score |*e*| as $\frac{1}{6}\sum_{i=1}^{6}e_{i}$. It is important to note that, this score is a conservative choice among other possible scoring options. This is because the average can include mapping scores of subnetworks with very low similarities from the original domain of SubMAP. This can underestimate the correct mapping score of |*e*| and hence degrade the correlation of compressed domain and original domain mapping scores. Overall, for each mapping in compressed domain with a score |*e^c^*| and we calculate the corresponding score |*e*| in the original domain using this average score.

Table 2 summarizes the correlation values found from a set of 3600 alignments (400 alignments for each parameter combination of *k *∈ {1, 2, 3} and *c *∈ {1, 2, 3}). We calculate the correlation of each query with the alignment that has the same *k *value but is in the original domain (i.e., *c *= 0). Table 2 shows the average correlation values of these 400 alignments for each *k *value, *c *value combination. The first column indicates that the alignment found by using only one compression level is highly similar to the alignment found by directly using the base method. Combining this with the running time gain in Figure 3(a) for *c *= 1, we can strongly argue that compression by one level not only provides significant improvement in running time but also accurately captures very high percentage of the original alignment results which makes it very useful for practical purposes. The accuracy measured in terms of correlation drops to 0.57 on the average when we perform the second level of compression and to 0.51 for the third level.

**Table 2:** Correlation of the mapping scores found with and without compression

*k/c*	1	2	3
1	0.89	0.56	0.53
2	0.85	0.58	0.50
3	0.84	0.57	0.49

We calculate the Pearson's correlation coefficient between the two sets of score values one from SubMAP (without compression) one from our framework (with compression) and report it as an indicator of the accuracy of alignment results of our framework for different parameter settings.

*These results suggest that we can almost always use one level of compression to benefit from a high performance gain without losing much accuracy in terms of the alignment results. For c *= *2 and c *= *3, even though the accuracy of the results are significantly better than random, such compression levels should be used with caution if the accuracy of the alignment is the main concern*.

## Conclusions

In this paper, we considered the problem of aligning two metabolic networks particularly when both of them are too large to be dealt with using existing methods. To solve this problem, we developed a framework that scales the size of the metabolic networks that existing methods can align significantly. Our framework is generic as it can be used to improve the scalability of any existing network alignment method. It has three major phases, namely the *compression phase*, the *alignment phase *and the *refinement phase*. For the first phase, we developed an algorithm which transforms the given metabolic networks to a compressed domain where they are summarized using much fewer nodes, termed supernodes, and interactions. In the second phase, we carried out the alignment in the compressed domain using an existing method, SubMAP, as the base alignment algorithm. In the refinement phase, we considered each individual mapping of *supernodes *one by one. Each such mapping corresponds to a smaller instance of network alignment problem. For each of these mappings, we solved the alignment problem using SubMAP as our base method. Our experiments on the metabolic networks extracted from the KEGG pathway database demonstrate that our compression method reduces the number of reactions by almost half at each level of compression. As a result of this compression, we observe that SubMAP coupled with our framework can align twice or more as large networks as its original version can with the same amount of resources. Our results also suggested that the alignment obtained by only one level of compression benefits from a significant performance gain while capturing the original alignment results with very high accuracy. We believe that this paper takes an important step in scaling the metabolic network alignment with subnetwork mappings to organism-wide networks, and thus, can have great impact on making the existing network alignment methods more useful for domain scientists.

## Methods

In this section, we describe the method we develop to compress the query networks and the overall framework for aligning networks in this compressed domain. Before going into detail, it is important to state that we are using a reaction-based model for representing metabolic networks throughout this paper. Formally, we represent a metabolic network with *P *= (*V*, *E*) where *V *is the set of all reactions of the network and *E *is the set of directed edges between them. An edge *e_ij _*∈ *E *exists if and only if the reaction *vi *has at least one output compound which is an input for the reaction *v_j_*. In the following, we first describe our compression method. We use the shorthand notation *MDS *(minimum degree selection) to refer to this method in the rest of the paper. We, then, prove the optimality of *MDS *under certain conditions and provide an upper bound for the number of compressions that can be missed by this method with respect to the optimal compression. Next, we give a brief overview of the base alignment method that we use in this paper and explain in detail the two remaining phases of our alignment framework. We provide our analysis on the computational complexity of the overall method and conclude the methods section by answering two questions related to performance characteristics of this method.

### Minimum degree selection (*MDS*) method

**Figure 5:**
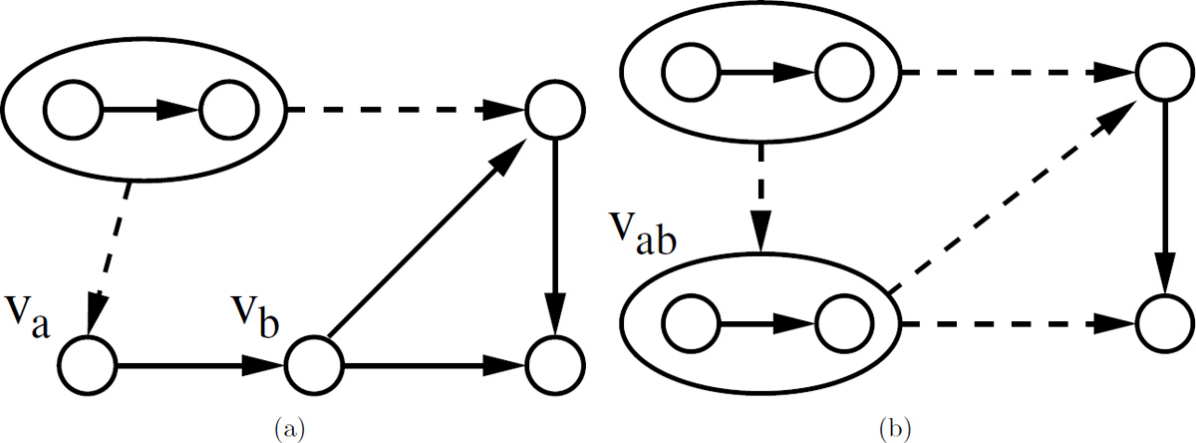
**One compression step of the *MDS *method.** Small circles represent reactions and big circles represent supernodes that result from earlier steps of compression. A solid arrow represents an edge between two non-compressed nodes in the current compression level. A dashed arrow denotes an edge between a supernode and another node in the network. While calculating the degrees of the non-compressed nodes, only the solid arrows are taken into account. (a) The state of network *P *during compression level *x *before the *i*th intermediate step (i.e., $P_{i-1}^{x}$). The node with the minimum degree is denoted with *v_a _*and its first neighbor is denoted with *v_b_*. (b) The state of this network after the *i*th compression step (i.e., $P_{i}^{x}$). We denote the node resulted from the compression at this step with *v_ab_*.

Let *P *= (*V*, *E*) be the reaction-based representation of a metabolic network and *c *denote the user specified parameter for the desired level of compression. For *x *= 1, ..., *c*, we denote the compressed form of *P *after *x *compression levels with *P^x ^*= (*V ^x^*, *E^x^*). To simplify our notation, we assume that *P*^0 ^= *P*. We construct *P^x ^*from *P^x ^*^- 1 ^for each *x *= 1, ..., *c*. Each *v *∈ *V ^x ^*is either a node from *V *^*x *- 1 ^or a supernode that contains two nodes of *V *^*x *- 1^. In summary, we construct *V ^x ^*from *V *^*x *- 1 ^in a number of consecutive steps. At each step, we choose a pair of connected nodes in *V *^*x *- 1 ^that are not compressed in earlier steps of the current compression level. We then merge this node pair into a supernode and add it to *V ^x^*. We repeat these steps until there is no such node pair in *V *^*x *- 1^. Assume that the number of such steps is *t *for compression level *x*. We denote the state of the network after the *i*th step during the *x*th level of compression as $P_{i}^{x}=\left(V_{i}^{x},E_{i}^{x}\right)$ Figure 5 (b)). Note that, $V_{t}^{x}=V^{x}$ and $V_{i}^{x}\subseteq V^{x-1}\cup V^{x}$ for each i = 1, ..., *t *as the nodes of $V_{i}^{x}$ are either singleton nodes from *V *^*x - *1 ^or supernodes from *V ^x^*.

We are now ready to discuss how we compress *P*^*x *- 1 ^to get *P^x^*. We define the *degree *of a non-compressed node *v *in a given network as *deg*(*v*) = *indeg*(*v*) + *outdeg*(*v*), where *indeg*(*v*) (*outdeg*(*v*)) denotes the number of incoming edges from (out-going edges to) non-compressed nodes in the network. We say that two nodes in a network are neighbors if they are connected by at least one edge. We denote the set of neighbors of a node *v *with *N*(*v*). We start the compression by initializing $V_{0}^{x}=V^{x-1},E_{0}^{x},E^{x-1}$. Then, while there exists a non-compressed node with degree greater than zero at the current state of the network, say $P_{i-1}^{x}$, we apply the next step, the *i*th step, of compression to obtain $P_{i}^{x}$ from $P_{i-1}^{x}$. Figure 5 depicts the states of an example network before (Figure 5(a)) and after (Figure 5(b)) the *i*th step of compression. We start the *i*th step by selecting a node with minimum positive degree among the nodes in $V_{i-1}^{x}$. If there are more than one such node, we select the first one among them. In our example in Figure 5(a), the node with minimum degree is unique and is shown by *v_a_*. We use the term minimum degree as a shorthand for minimum positive degree to exclude singleton nodes. This way we ensure that *deg*(*v_a_*) *>*0 and *N *(*v_a_*) is non-empty. We select one such neighbor from *N*(*v_a_*), say *v_b_*. The only node in *N *(*v_a_*) in Figure 5(a) is denoted with *v_b_*. We, then, merge *v_a _*with *v_b _*to form the supernode *v_ab _*= {*v_a_*, *v_b_*}. Figure 5(b) illustrates this newly created node *v_ab_*. This is the only compression to be done at the *i*th compression step. Next, we create the new node set as $V_{i}^{x}=V_{i-1}^{x}\cup \left\{v_{ab}\right\}-\left\{v_{a},v_{b}\right\}$. For creating the edge set $E_{i}^{x}$, we initialize it to $E_{i-1}^{x}$ and remove all the incoming and out-going edges of *v_a _*and *v_b _*from it. Then, we insert an incoming edge to *v_ab _*from each node in $V_{i-1}^{x}-\left\{v_{a},v_{b}\right\}$, which has an out-going edge to either *v_a _*or *v_b _*in the previous edge set $E_{i-1}^{x}$. We insert out-going edges from *v_ab _*to other nodes in a similar manner. Figure 5 illustrates the changes in the edge set after creating *v_ab_*. Notice that for each *i *= 1, ..., *t*, the set $V_{i}^{x}$ contains a mixture of nodes and supernodes. After each such step, the size of the network decreases by one and the number of edges of the new network decreases at least by one. For instance in Figure 5, the number of nodes dropped from five to four and the number of edges dropped from six to five. The compression of *P*^*x-*1 ^to get *P^x ^*continues by applying another compression step until there are no more non-compressed nodes with positive degree.

The discussion above describes the intermediate compression steps of the *MDS *method to perform a single level of compression on a given network. Given a compression level *c*, for each level *x *= 1, ..., *c*, we apply the same compression steps on *P*^*x *- 1 ^= (*V *^*x *- 1^, *E*^*x *- 1^) by initially treating *P*^*x *- 1 ^as a non-compressed network with no supernodes. As a result of this process, after finishing the *x*th level of compression, the actual number of reactions that each node of *V ^x ^*can contain is assure to be in the interval [1, 2*^x^*]. The limitation on the number of reactions in each node allows the *MDS *method to respect and highly preserve the initial topology of the query networks. This is very important for the alignment as it makes significant use of the network topologies. Additionally, the bound on the number of reactions in each supernode translates to a uniform compression for both networks which limits the sizes of the smaller alignment problems we can encounter in the refinement phase. This allows us to keep under control the complexity and the running time of the refinement phase of our alignment framework.

### Optimality analysis for *MDS*

In the previous section, we described in detail the compression method (*MDS*) we use in our framework. Ideally, it is preferable to compress the given network as much as possible at each compression level. This is because smaller network size often implies smaller time and memory usage for the alignment. We say that a compression is *optimal *if the resulting compressed network contains the smallest number of nodes among all possible compressions with the restriction that each non-compressed node can be merged with at most one other non-compressed node at each compression level. We name the hypothetical optimal compression method that can achieve the best possible compression rate as *OPT*. In the rest of this section, we analyze the optimality of our *MDS *method under different conditions. We first consider each connected component of the input network that will be compressed separately and then integrate their results to generalize our analysis for networks with arbitrary topologies.

We start by introducing the notation we use in this section to handle networks with more than one connected component. Let *P *be a metabolic network with *r *connected components. We denote these components by $C_{1}=\left({\hat{V}}_{1},\;{Ê}_{1}\right),\;C_{2}=\left({\hat{V}}_{2},{Ê}_{2}\right),\;\ldots ,\;C_{r}=\left({\hat{V}}_{r},{Ê}_{r}\right)$, such that $P=\left({⋃}_{j=1}^{r}{\hat{V}}_{j},\;{⋃}_{j=1}^{r}{Ê}_{j}\right)$. Let $C=\left(\hat{V},Ê\right)$ be an arbitrary component of *P *and *^x ^represent the compressed form of *C *after *x *levels of compression using either the *MDS *method or *OPT *that achieves the optimal compression. We use * (star) as a generic symbol to avoid introducing new symbols for each compressed component in places where only their sizes are of relevance. We use *MDS*(*C*, *^x^), *OPT*(*C*, *^x^) to denote the total number of compression steps performed to transform *C *into its compressed form after *x *levels of compression by using the corresponding methods. Recall that each compression step reduces the network size by one. Thus, the bigger these values (*MDS*(*C*, *^x^) and *OPT*(*C*, *^x^)) the better they are in terms of compression rate. The first and second arguments in this notation can be any state of a connected component or a network at any point during the compression. For instance, $OPT\left(C_{i}^{x},{*}^{x}\right)$ denotes the number of compression steps taken by *OPT *starting from (*i *+ 1)th intermediate step of the *x*th level until the *x*th level of compression is completed.

In the following, we first prove that the *MDS *method makes an optimal choice in terms of which two nodes to compress at each compression step if there exists a node with degree one in the current state for a given component. We, then, show that if no node with degree one exists at a compression step taken by *MDS *can increase the size of the compressed component by at most one as compared to the one found by *OPT*. Finally, by aggregating the results from each component, for a given metabolic network *P *and a compression level *c*, we develop an upper bound on the size of the compressed networks obtained by *MDS *with respect to the size of network that can be obtained by the optimal method.

**Lemma 1 ***Let *$C=\left(\hat{V},Ê\right)$*denote a connected component of a given metabolic network P. Let *$C_{i}^{x}=\left({\hat{V}}_{i}^{x},{Ê}_{i}^{x}\right)$* denote the state of C after the ith step of the xth compression level. If there exists a node in *${\hat{V}}_{i}^{x}$* with degree one, then the compression step taken by the MDS method to create the next state *$C_{i+1}^{x}$*is optimal. Formally*,

(1) $$O\;PT\left(C_{i}^{x},{*}^{x}\right)=1+O\;PT\left(C_{i+1}^{x},\;{*}^{x}\right)$$

**Proof 1 ***We prove (1) by contradiction in two parts:*

*Part 1. *$O\;PT\left(C_{i}^{x},\;{*}^{x}\right)≮1+O\;PT\left(C_{i+1}^{x},\;{*}^{x}\right)$

*Part 2. *$O\;PT\left(C_{i}^{x},\;{*}^{x}\right)≯1+O\;PT\left(C_{i+1}^{x},\;{*}^{x}\right)$

*The first part (i.e.*, ≮*) is trivial. The number of compression steps of OPT after performing one step of compression cannot be larger than the number before performing this step, otherwise the solution of *$OPT\left(C_{i}^{x},{*}^{x}\right)$*cannot be optimal. This leads to a contradiction, hence proves Part 1*.

*To prove the second part (i.e.*, ≯*), it is important to recall how the MDS method progresses given the state $C_{i}^{x}$ at which there exists at least one node v_a _with deg*(*v_a_*) = 1*. This method picks v_a_. The node v_a _has exactly one non-compressed neighbor, say v_b_. Thus, MDS merges them to create the supernode v_ab _(see *Figure 5*). We complete the proof by considering two cases. In the first case the OPT method merges v_a _and v_b _while compressing *$C_{i}^{x}$*. In this case, we can assume that OPT takes this step as its next step in compressing *$C_{i}^{x}$*, since a fixed compressed network can be obtained by arbitrarily shuffling the order of intermediate steps. Therefore, if v_a _and v_b _are compressed at any point in the optimal method, then the optimal solution for *$C_{i+1}^{x}$*, which is created by applying the MDS method on *$C_{i}^{x}$ has exactly $O\;PT\left(C_{i}^{x},\;{*}^{x}\right)-1$ compressions. Hence, $O\;PT\left(C_{i}^{x},\;{*}^{x}\right)=l\;+\;O\;PT\left(C_{i+1}^{x},\;{*}^{x}\right)$and $O\;PT\left(C_{i}^{x},\;{*}^{x}\right)≯1+O\;PT\left(C_{i+1}^{x},\;{*}^{x}\right)$

*In the second case v_a _and v_b _are not merged together in the optimal solution. This case implies v_a _is left as a singleton at the end of the xth level as deg*(*v_a_*) = 1*. Then, the network that results after removing v_a _and all the edges connected to it can have at most *$OPT\left(C_{i}^{x},{*}^{x}\right)$ compressions until the end of the xth level since otherwise it contradicts with the optimality of MDS. This shows that the number of compressions that can be achieved when v_a _is left as a singleton cannot be greater than one plus $OPT\left(C_{i+1}^{x},\;{*}^{x}\right)$. Thus, $O\;PT\left(C_{i}^{x},\;{*}^{x}\right)≯1+O\;PT\left(C_{i+1}^{x},\;{*}^{x}\right)$* and combining it with the first part (i.e.*, ≮) we get $O\;PT\left(C_{i}^{x},\;{*}^{x}\right)=1\;+\;O\;PT\left(C_{i+1}^{x},\;{*}^{x}\right)$. □

**Lemma 2 ***Let $C=\left(\hat{V},Ê\right)$ denote a connected component of a given metabolic network P. Let *$C_{i}^{x}=\left({\hat{V}}_{i}^{x},{Ê}_{i}^{x}\right)$*denote the state of C after the ith step of the xth compression level. If the node with minimum degree in *${\hat{V}}_{i}^{x}$*has degree greater than one, then the compression step taken by MDS to create the next state *$C_{i+1}^{x}$*can lead to a network that has size at most one larger than the compressed network that is obtained from the state *$C_{i}^{x}$*by OPT. Formally*,

(2) $$O\;PT\left(C_{i}^{x},\;{*}^{x}\right)\leq 2+O\;PT\left(C_{i+1}^{x},\;{*}^{x}\right)$$

**Proof 2 ***Let v_a _be the first node in the list of minimum degree nodes in *${\hat{V}}_{i}^{x}$*. From the assumption we know deg*(*v_a_*) *>*1 *and hence it has at least one non-compressed neighbor node of v_b _that also has deg*(*v_b_*) *>*1*. Without loss of generality assume that the MDS method merges v_a _and v_b _to create the supernode v_ab _at the compression step from *$C_{i}^{x}$*to *$C_{i+1}^{x}$*. This step can prevent at most one neighbor of v_a_, say v_c_, and at most one neighbor of v_b_, say v_d_, to be merged with the corresponding node in later steps. Notice that v_c _and v_d _are not necessarily distinct. The MDS algorithm can also merge v_c _and v_d _in the next steps if they are also neighbors though we do not know it for sure at this point. This results in either one compression or two compressions using only the four nodes v_a_, v_b_, v_c _and v_d _by the MDS method. Next, we calculate the number of compression steps that the OPT method can take for compressing these four nodes. There are three cases to consider:*

Case 1. The *OPT *method merges *v_a _*with *vb *at any point during the *x*th level of compression. This case is equivalent to merging v_a _with v_b _in the next step by MDS and then compressing the rest of the network by O PT. In other words, MDS already takes the optimal compression step. Hence, $O\;PT\left(C_{i}^{x},\;{*}^{x}\right)=1\;+\;O\;PT\left(C_{i+1}^{x},\;{*}^{x}\right)\leq 2+O\;PT\left(C_{i+1}^{x},\;{*}^{x}\right)$.

Case 2. The *O PT *method merges *v_a _*with *v_c _*at any point during the *x*th level of compression. *The worst case scenario for the MDS method in this case is when v_c _is not connected to v_d _and the OPT method merges v_b _with v_d _in a later step. This way the OPT method optimally compresses four nodes down to two supernodes, namely v_ac _and v_bd_. On the other hand the MDS method creates a single supernode, v_ab_, and the nodes v_c _and v_d _remain as singleton However, even for this worst case, the MDS method prevents only one compression step to take place with respect to O PT. Hence, $O\;PT(C_{i}^{x},\;{*}^{x}))\leq 2+O\;PT(C_{i+1}^{x},\;{*}^{x})$*.

Case 3. The *O PT *method merges *v_b _*with *v_d _*at any point during the *x*th level of compression. *We can prove this similar to *Case 2 *by the symmetry*.

□

Using lemmas 1 and 2, Theorem 1 develops an upper bound on the number of compression that can be missed by *MDS *with respect to the optimal compression.

**Theorem 1 **(OPTIMALITY BOUND FOR MDS) *Let P be a metabolic network with r connected components $C_{1}=\left({\hat{V}}_{1},{Ê}_{1}\right),\;\ldots ,\;C_{r}=\left({\hat{V}}_{r},{Ê}_{r}\right)$ such that $P={⋃}_{j=1}^{r}C_{j}$ and c be a positive integer given as the desired number of compression levels. Let *$C=\left(\hat{V},Ê\right)$*denote an arbitrary connected component of P. Also, let s represent the number of intermediate steps for which no non-compressed nodes with degree one is found during the compression from P to P^c ^by the MDS method*.

Then, each of the following statements hold:

*1*. *O PT *(*C*^*x *- 1^, *^x^) ≤ 2 *MDS *(*C*^*x *- 1^, *^x^) *for × *= *1*, ..., *c*.

*2*. *O PT *(*P*, *^c^) ≤ *s + MDS *(*P*, *^c^)

*3*. *O PT *(*P*, *^c^) ≤ *min*{2 *MDS *(*P*, *^c^)*, s + MDS *(*P*, *^c^)}.

**Proof 3 ***1. This part follows from Lemma 1 and 2. Lemma 1 states the case when MDS method is equivalent to OPT. Lemma 2 gives an upper bound on the number of compression steps that MDS can miss. The worst case is when the boundary condition of Lemma 2 holds for each step of the xth compression level for C*^*x *- 1^*. In this case, the number of steps taken by the OPT method while compressing C*^*x *- 1 ^*is two times the number for the MDS method*.

*2*. *This part also follows from Lemma 1 and 2. Throughout the compression of the entire network P by c levels, each step of the MDS method that satisfies the condition in Lemma 2 can decrease the number of possible merge operations by one with respect to OPT. By simply counting these steps, at the end of the execution of the MDS method we can give the upper bound s+ MDS *(*P*, *^c^) *on the number of optimal compressions O PT *(*P*, *^c^).

*3*. *Part 2 shows that O PT*(*P*, *^c^) ≤ *s+ MDS *(*P*, *^c^)*. It is only necessary to show O PT*(*P*, *^c^) ≤ 2 *MDS *(*P*, *^c^)*. Part 1 proves this result for a single connected component C for the xth compression level. P is given as ${⋃}_{j=1}^{r}C_{j}$ before the first level of compression. We know by Part 1 that O PT *(*C*, *^1^) ≤ 2 *MDS*(*C*, *^1^)*. Summing this up for all j from 1 to r, we get OPT*(*P*, *^1^) ≤ 2 *MDS*(*P*, *^1^). This equation holds for each compression level x from 1 to c. Summation over x gives $\sum_{x=1}^{c}\left(OPT\left(P^{x-1},\;{*}^{x}\right)\right)\leq \sum_{x=1}^{c}MDS\left(P^{x-1},\;{*}^{x}\right)$. Hence, we prove OPT(*P*, *^c^) ≤ 2 *MDS*(*P*, *^c^).

□

Another way of interpreting Theorem 1 is to transform it to an upper bound on the size of the compressed network generated by *MDS *in terms of the one that can be obtained by *OPT*. By carrying out this transformation, we answer the question we pointed out in the introduction which is "How far is our compression method from the optimal compression?". We do this as follows. Let *P *be a network of size *n*. Given compression level *c*, let us represent the number of compressions steps of the *O PT *method with *θ *= *O PT *(*P*, *^c^). Also, let *n_O PT _*and *n_MDS _*denote the sizes of the compressed networks obtained by the *OPT *and *MDS *methods respectively. By the bound given in Theorem 1, we know that $MDS\left(P,\;{*}^{c}\right)>=\left\lceil \frac{\theta }{2}\right\rceil$. Therefore, we can write *n_O PT _*= *n *- *θ*. and $n_{MDS}\leq n-\left\lceil \frac{\theta }{2}\right\rceil$. Also, we know by definition that $\theta \leq \sum_{x=1}^{c}\left\lfloor \frac{n}{2^{x}}\right\rfloor$. Using this inequality, we get:

(3) $$n_{OPT}\geq n-\overset{c}{\underset{x=1}{\sum }}\left\lfloor \frac{n}{2^{x}}\right\rfloor ,n_{MDS}\leq n-\left\lceil \overset{c}{\underset{x=1}{\sum }}\left\lfloor \frac{n}{2^{x+1}}\right\rfloor \right\rceil$$

If we examine the ratio $\frac{n_{MDS}}{n_{O\;PT}}$, for *c *= 1 we get $\frac{n_{MDS}}{n_{O\;PT}}\leq \frac{3}{2}$ for arbitrary *n *(details omitted). This demonstrates that after one level of compression, the size of the compressed network found by our method is at most 1.5 times the size of the optimal network. For *x *= 1, 2, ..., *c*, this ratio is proportional with (1.5)*^x^*. We can also use the bound on number of compression steps given in the second statement of Theorem 1 to gather a similar upper bound on the size of the compressed network found by *MDS*. The tighter of these two upper bounds on the network size can be calculated during the execution of the *MDS *method and reported as an indicator of how much room is left for improving the compression.

### Alignment framework

We described the first phase, namely the compression phase in detail in previous sections. Here, we first summarize the base alignment method, SubMAP [10], we use in our framework. Then, we explain the two remaining phases of our framework, namely the alignment phase and the refinement phase. The alignment phase follows the compression phase and utilizes the base method to find an alignment in compressed domain. The refinement phase applies the base method on the mappings found in previous phase to further refine the alignment results. After describing all the phases, we analyze the complexity of each phase and combine them to obtain the complexity of the entire framework. Then, we examine the characteristics of the queries to determine which are likely to benefit from compression during the alignment to answer the question of "When should we compress?" Last, we provide a guideline for selecting the compression level that is expected to give the best performance gain reached by our framework with respect to the base alignment method.

### Overview of SubMAP

Here, we take a small detour and explain SubMAP, a recent method for aligning metabolic networks when they are not compressed. We pick SubMAP method for its high accuracy and biological relevance as it considers subnetworks of the given networks during the alignment. A *subnetwork *of a network is a subset of the reactions of that network such that the induced undirected graph of this subset is connected. Given two metabolic networks *P *= (*V*, *E*) and $\bar{P}=\left(\bar{V},Ē\right)$ and a positive integer *k*, SubMAP aims to find a set of mappings between the reactions of *P *and $\bar{P}$ with the largest similarity score, such that: (i) Each reaction in $P\;\left(\bar{P}\right)$ can map to a subnetwork of $\bar{P}\;\left(P\right)$ with at most *k *reactions (ii) Each reaction of *P *and $\bar{P}$ can appear in at most one mapping.

The first step of SubMAP is to create the set of all possible subnetworks of size at most *k *for each query network. We denote the number of these subnetworks for *P *and $\bar{P}$ with *N_k _*and *M_k _*respectively. The second step of SubMAP is to calculate pairwise similarities between each pair of these subnetworks one from *P *and one from $\bar{P}$. Each subnetwork consists of reactions and each reaction is defined by its input and output compounds (i.e., substrates and products) and the enzymes that catalyze it. Therefore, we measure the pairwise similarities between subnetworks using reaction similarities which in turn are defined by the similarities of the components of these reactions. For more details of this similarity score we refer the reader to Ay *et al*. [10].

The step that dominates the time and space complexity of SubMAP is the third step. The aim of this step is to create a similarity score that combines pairwise similarities with the topological similarity of the networks. A data structure named the *support matrix *is created for this purpose. The size of this matrix is quadratic in terms of the number of subnetworks of both query networks. In other words, the support matrix requires *O *(*N_k_*^2 ^*M_k_*^2^) space. This complexity is very important as it is the dominating factor in the overall time and space complexity of SubMAP. The next two steps of the algorithm are to combine topological similarity with pairwise node similarities and to extract the alignment as a set of subnetwork mappings of *P *and $\bar{P}$.

### Alignment phase

The SubMAP method described above aligns the networks *P *= (*V*, *E*) and $\bar{P}=\left(\bar{V},Ē\right)$ in their original form. Our framework first compresses each of these networks to reduce their sizes and then aligns the compressed networks instead of *P *and $\bar{P}$. In this section, we explain how we align the compressed networks *P^c ^*and ${\bar{P}}^{c}$ that are in the compressed domain of level *c *using SubMAP with a given parameter *k*.

Let us first consider *P^c ^*= (*V ^c^*, *E^c^*). Each node *v_a _*in *V ^c ^*is a supernode of the reactions in *V *. Also, by the working of our compression method, we know that each supernode *v_a _*contains at most 2*^c ^*reactions. An edge from the node *v_a _*to the node *v_b _*exists in *E^c ^*if and only if at least one reaction in *v_a _*has an edge to one reaction in *v_b _*in *E*. The same arguments hold for the other network ${\bar{P}}^{c}$ as well. To align these compressed networks, we consider their nodes, which are supernodes of reactions, as if they are the reactions of the metabolic networks *P^c ^*and ${\bar{P}}^{c}$. This way, we can directly apply SubMAP to align these networks. As far as the operation of the SubMAP method is concerned, this is no different than aligning two networks that are identical to these networks but are in the original domain. The difference is in the interpretation of the intermediate steps and the form of the mappings found by the alignment. For instance, for the first step of SubMAP, we enumerate the reaction subnetworks of size at most *k *in the original domain, whereas in the compressed domain we enumerate the subnetworks of supernodes where each supernode can contain more than one reaction and the number of such supernodes in one subnetwork is at most *k*. Similarly, we calculate the pairwise similarity, the support matrix and the conflict graph for the subnetworks of supernodes (i.e., nodes of *V ^c^*) instead of subnetworks of reactions (i.e., nodes of *V *). The resulting alignment gives us a set of mappings between the subnetworks of *P^c ^*and ${\bar{P}}^{c}$. We can think of these mappings as a high level view of the alignment between the networks *P *and $\bar{P}$. For instance, from Figure 1(f) one can immediately see that the resulting alignment will map node *a *either to node *a' *or node *b' *and that these are the only options for node *a *which is imposed by the higher level supernode mapping (*a*, *b *- *a'b'*). In the next phase, we consider each of these supernode mappings as smaller instances of the alignment problem and solve them to obtain a more refined alignment of *P *and $\bar{P}$.

### Refinement phase

Each mapping found by the alignment phase is a subnetwork pair where one is from *P^c ^*and the other is from ${\bar{P}}^{c}$. The mappings found by SubMAP can have up to *k *nodes in one subnetwork and only one node in the other. If we denote a subnetwork of *P^c ^*with $R_{i}^{c}$ and a subnetwork of ${\bar{P}}^{c}$ with ${\bar{R}}_{j}^{c}$, the resulting mappings of the alignment phase will be in the form $\left(R_{i}^{c},\;{\bar{R}}_{j}^{c}\right)$. We can assume, without loss of generality, for this specific pair that $R_{i}^{c}$ contains up to *k *nodes of *P^c ^*and ${\bar{R}}_{j}^{c}$ contains a single node of ${\bar{P}}^{c}$. Each node contained in either of these subnetworks is a supernode that contains either one node or two nodes and an edge between them in the previous level of compression, namely the (*c *- 1)th level. For both $R_{i}^{c}$ and ${\bar{R}}_{j}^{c}$, we decompress their nodes by one level by retrieving the connectivity between these nodes in the (*c *- 1)th compression level that was encapsulated in the *c*th level. This decompression results in at most 2*k *nodes from (*c *- 1)th level for $R_{i}^{c}$ and at most 2 nodes from (*c *- 1)th level for ${\bar{R}}_{j}^{c}$. We then recursively align these smaller networks generated from $R_{i}^{c}$ and ${\bar{R}}_{j}^{c}$ by using SubMAP until the original domain (i.e., *c *= 0) is reached. At the (*c *- *x*)th recursive step, the sizes of two networks to be aligned can be at most *k *2*^x ^*for one network and 2*^x ^*for the other.

Figure 1(f) illustrates this on a concrete example. The network on the left has two supernodes (i.e., (*a*, *b*) and (*e*, *d*)) each containing two nodes with an edge between them and one supernode (i.e., (*c*)) which contains only one node from the previous level of compression. The one on the right has two supernodes with two nodes in each. To understand how decompression by one level works, we can focus on the supernode mapping (*e*, *d*) - (*c'*, *d'*) which is found in compression level one. We can think of decompression as removing the circles that surround these supernodes to get back the connectivity within their nodes in the previous compression level. In our case, this leads to the small networks *d *→ *e *and *c' *→ *d'*. We align these small networks recursively using SubMAP and report their final alignment in only one recursive call since the compression level is only one for this case. Also, since *k *= 1 is used for the ease of this example, the sizes of the networks, in terms of the nodes in original domain, on each side are at most 2 for the recursive call from *c *= 1 as can be seen from Figure 1(f) (i.e., *k *2*^c ^*= 2*^c ^*= 2 for *k *= *c *= 1).

### Complexity analysis

Having finished the discussion of all the three phases, now we can analyze the overall complexity of our framework. We start from the first phase which is compression of the input networks *P *and $\bar{P}$ by *c *levels. We first calculate the complexity of the first compression level for the network *P *with size *n*. At each compression step, *MDS *first searches for a minimum degree node. Once it finds this node, it picks one of its neighbor nodes and merges these two nodes. After this merging, it updates the degrees of all the neighbors of each of the merged nodes. The first two of these operations take *O *(*log n*) time if proper data structures are used and the last one can take *O *(*n*) in the worst case. Since the size of network *P *is *n*, there can be at most $\left\lfloor \frac{n}{2}\right\rfloor$ compression steps during the first level of compression. Hence, the complexity of the compression for the first level is *O *(*n*^2^). Since the input sizes of this level is larger than all the next levels, we can safely assume that each of these next levels also take *O*(*n*^2^) and the complexity of compression by *c *levels is therefore *O *(*cn*^2^). Even though this is not a tight bound, it is sufficient at this point for the complexity of the next two phases will dominate it. Since we compress both networks, the overall complexity for the compression phase is:

(4) $$O\left(c\left(n^{2}+m^{2}\right)\right).$$

For the analysis of the next phases, we make two assumptions both of which are supported by experimental evidence on the topological properties of metabolic networks. Our first assumption is that at each level of compression our method reduces the network size by half. In other words, if the sizes of our query networks are *n *and *m*, then the sizes of the compressed networks after *c *levels by the *MDS *method are $n_{MDS}=\left\lceil \frac{n}{2^{c}}\right\rceil$ and $m_{MDS}=\left\lceil \frac{m}{2^{c}}\right\rceil$ respectively. This is mainly because metabolic networks contain many nodes with low degrees [27]. Our experiments on a large dataset of networks summarized in Table 1 supports this as well. The second assumption is that the number of subnetworks is a constant multiple of the network size for small *k *values. In other words, *N_MDS _*= *α *(*k*) *n *and *M_MDS _*= *β *(*k*) *m *where *α *(*k*) and *β *(*k*) are functions of *k *but are independent of *n *and *m *respectively. Our earlier analysis in Ay *et al*. [10] demonstrated that the number of subnetworks for *k *= 3, which is the largest *k *value we use here, is in the order of 5|*V *| for a large set of metabolic networks.

We are now ready to analyze the complexity of the second phase which is the alignment phase. By the first assumption, we know that the sizes of *P^c ^*and ${\bar{P}}^{c}$ are $n_{MDS}=\left\lceil \frac{n}{2^{c}}\right\rceil$ and $m_{MDS}=\left\lceil \frac{m}{2^{c}}\right\rceil$ respectively. By the second, we have the number of subnetworks of these networks as *N_MDS _*= *α *(*k*) *n *and *M_MDS _*= *β *(*k*) *m *for a given *k*. Also, we know that the complexity of SubMAP is quadratic in terms of *N_MDS _*and *M_MDS_*. Therefore, the complexity of the second phase is:

(5) $$O\left(\frac{\alpha {\left(k\right)}^{2}\beta {\left(k\right)}^{2}n^{2}m^{2}}{2^{4c}}\right).$$

The complexity of the refinement phase has two factors in it. The first one is the number of mappings found by the alignment phase. Since we know that SubMAP allows each node of both networks to be reported in at most one mapping, we have a trivial upper bound on the number of possible mappings in terms of *n *and *m*. The biggest number of mappings is reported when all the subnetworks of both networks are singletons. In this case, the number of reported mappings is the minimum of *n *and *m*. We can assume without loss of generality that *n < m *and hence this number is *O *(*n*). The second factor is the sizes of each of these *O*(*n*) smaller alignment problems that needs to be solved by SubMAP again to refine the mapping results. As we discussed in the refinement phase, the sizes of the networks created by decompressing the mapped subnetworks by one level are at most *k *2*^c ^*on one side and at most 2*^c ^*on the other. The number of subnetworks that can be created from these networks are *α *(*k*) *k *2*^c ^*and *β *(*k*) 2*^c ^*for the corresponding sides. Therefore, each mapping can be refined by decompressing and applying SubMAP which is *O *(*α *(*k*)^2 ^*k*^2 ^2^2*c *^*β *(*k*)^2 ^2^2*c*^). We do this refinement for *O *(*n*) times in the worst case, hence the complexity of the refinement phase is:

(6) $$O\left(\alpha {\left(k\right)}^{2}\beta {\left(k\right)}^{2}nk^{2}2^{4c}\right).$$

Combining the results of Equations 4, 5 and 6, we can see that the overall complexity of our method is determined by the second or the third phase depending on the value of *c*. For small values of *c *and *k *such as 1, 2 and 3, the second phase dominates the overall complexity. Larger values of *c *results in a costlier refinement phase and a less expensive alignment phase. Very large values of *k *imply exponentially many subnetworks in which case the above complexity analysis would not hold and the alignment problem may become intractable with or without compression.

### When should we compress?

We discussed the potential of our framework improving the scalability of existing network alignment methods. However, there can be cases when the compression results in such network topologies which would enforce the alignment method to reach its worst case performance. In this section, we want to analyze when performing the alignment in compressed domain is the better alternative. For this purpose, we devise a criterion that is inspired by the results of a large number of network alignments that are done by both of the methods. We find that the gain/loss in running time is highly dependent on the number of all possible subnetworks of compressed and non-compressed networks. The numbers of these subnetworks can be determined in advance to the alignment. By formulating a criterion in terms of these numbers, we can make a decision between the two algorithms before actually performing an alignment.

Figure 4 illustrates the results for 3600 alignments performed by both of the methods on a wide range of network sizes with all possible combinations of *k *and *c *values. The x-axis show the running time of SubMAP minus the running time of our framework. The bigger this value is the better improvement we get from our framework. The y-axis shows the ratio $y=\frac{N_{k}^{c}M_{k}^{c}}{N_{k}M_{k}}$ where *N_k_*, *M_k _*denote the numbers of all subnetwork of *P *and $\bar{P}$ and $N_{k}^{c}$, $M_{k}^{c}$ denote the numbers of all subnetwork of the compressed networks *P^c ^*and ${\bar{P}}^{c}$. The dashed line passing from *y *= 0.5 visualizes our criterion. If the above ratio is below 0.5, then the number of all possible subnetworks generated by the compressed alignment is less than the half of this number for the original alignment. Very large portion of the alignments (97%) satisfying this criterion shows improvement in running time if compression is used. For the upper part of 0.5, only a small portion of these alignments (10%) shows improvement. Considering the overhead of refinement phase and the compression phase, this result is expected. These results strongly suggest that the answer to the question "When should we compress?" is "when $\frac{N_{k}^{c}M_{k}^{c}}{N_{k}M_{k}}\leq 0.5$".

### How much should we compress?

In this section, we provide a guideline for selecting a value for compression level *c *that results in the minimum expected running time, among other possible values, for our framework to align the query networks with for a given *k*. We make extensive use of the computational complexity results we discussed before in the proof of the below theorem which formulates the optimal *c *for a given *k *value and the two query networks with sizes *n *and *m*. This theorem answers the question "What is the right amount of compression that we need to use in order to minimize the running time of our framework?".

**Theorem 2 **(OPTIMAL LEVEL OF COMPRESSION) *Let P *= *(V, E), $\bar{P}=\left(\bar{V},Ē\right)$ be two metabolic networks with sizes n and m respectively, and k be a given positive integer. Assume without loss of generality that n < m. Then, the compression level c that gives the optimal compression is:*

(7) $$c=\frac{\log_{2}\left(nm^{2}k^{-2}\right)}{8}.$$

**Proof 4 ***Given P and *$\bar{P}$*, we want to find c value such that the difference between the complexity of applying SubMAP to align these networks in their original domain for a given k and the complexity of using our framework that aligns P with *$\bar{P}$*in compressed domain for the same k value is maximum. We omit the constant factors and use the algorithmic complexity as the cost of alignment. Under this assumption, the cost of aligning two networks with sizes n and m with SubMAP in the original domain for a given k value is:*

(8) $$\alpha {\left(k\right)}^{2}\beta {\left(k\right)}^{2}n^{2}m^{2}$$

*For our framework, this cost can be determined from the complexities of three different phases given by the Equations (4), (5) and (6) (see main article for these equations). As discussed, the dominating factors in the complexity are the last two phases (i.e., Equation (5) and Equation (6)). Therefore, we write the total cost of aligning P with *$\bar{P}$*in the compressed domain c, for a given k value as:*

(9) $$\frac{\alpha {\left(k\right)}^{2}\beta {\left(k\right)}^{2}n^{2}m^{2}}{2^{4c}}+\alpha {\left(k\right)}^{2}\beta {\left(k\right)}^{2}nk^{2}2^{4c}$$

*Our aim is to maximize (8) *- *(9) with respect to c. We know that this difference is negative (i.e., alignment in compressed domain is costlier) when c *≥ *n (assuming n < m as stated in the Theorem) or when c *= 0 *due to the overhead of compression and/or refinement phases. We also know that, for c *= 1 *this difference is positive as compression by one level always results in less costlier alignments compared to no compression. Therefore, if there is an extrema of (8) *- *(9) with respect to c for c *∈ (0, *n*)*, then this extrema is a maxima meaning that the difference (8) *- *(9) is maximum at that point. We calculate this maxima by derivation of (8) - (9) with respect to c and setting it to zero as:*

(10) $$\begin{array}{ll} \frac{\partial \left(\left(1\right)-\left(2\right)\right)}{\partial c} & =0\;\\ \frac{\partial \left\{\alpha {\left(k\right)}^{2}\beta {\left(k\right)}^{2}n^{2}m^{2}-\alpha {\left(k\right)}^{2}\beta {\left(k\right)}^{2}n^{2}m^{2}2^{-4c}-\alpha {\left(k\right)}^{2}\beta {\left(k\right)}^{2}nk^{2}2^{4c}\right\}}{\partial c} & =0\;\\ 4\log\left(2\right)2^{-4c}\alpha {\left(k\right)}^{2}\beta {\left(k\right)}^{2}n^{2}m^{2}-4\log\left(2\right)2^{4c}\alpha {\left(k\right)}^{2}\beta {\left(k\right)}^{2}nk^{2} & =0\;\\ 2^{-4c}nm^{2}-2^{4c}k^{2} & =0\;\\ 2^{8c} & =nm^{2}k^{-2}\;\\ c & =\frac{\log_{2}\left(nm^{2}k^{-2}\right)}{8}\;\\ & \; \end{array}$$

□

The value obtained from the above discussion is not necessarily an integer. We suggest using the nearest integer to this value as the number of compression levels in our alignment. Next, we want to give a few examples for to see what Theorem 2 implies in practice. Assume we have two networks with sizes *n *= 100, *m *= 100 and we want to align them using our framework for *k *= 2. Plugging these number in Equation 7, we get:

$$c=\frac{\log_{2}\left(250000\right)}{8}=\frac{17.93}{8}≅2.24$$

If we round this to the nearest integer, the Equation 7 suggests that we use two levels of compression for this alignment problem to be able to get the largest gain in running time. We can carry the calculations similarly for a bigger set of inputs *n *= *m *= 1000 and *k *= 3 which gives around 3.34, suggesting three levels of compression is likely to provide the best running time improvement for this instance.

However, it is important to note that depending on how much of a tradeoff is desired between the running time gain and the alignment accuracy, the user can always use smaller (or bigger) *c *values than the ones suggested here. Also, the above calculated values are only expected to provide the best running time improvement with respect to the original alignments running time. If the size of the query is orders of magnitude bigger than the original algorithm can handle, then it is likely that the framework we propose here to also fail to perform the alignment.

## List of abbreviations

*P *= (*V*, *E*), $\bar{P}=\left(\bar{V},Ē\right)$: Query metabolic networks; *V*, $\bar{V}$: Sets of all reactions of the query networks; *r_i _*∈ *V*, ${\bar{r}}_{j}\in \bar{V}$: Reactions of the query networks; *n *= |*V *|, $m=|\bar{V}|$: Sizes of the query networks; *c*, 2*^c^*: Compression level and compression rate; *P^c ^*= (*V ^c^*, *E^c^*): *P *after *c *levels of compression; $C_{i}=\left({\hat{V}}_{i},{Ê}_{i}\right)$: A connected component of network *P*; *N*(*v_a_*), *deg*(*v_a_*): The set of neighbors and degree of node *v_a_*; |*v_a_*|: Number of reactions that are contained in *v_a_*; *v_ab _*: A supernode containing the nodes *v_a _*and *v_b_*; *k*: Parameter for the largest subnetwork size; ${ℛ}_{k},{\bar{ℛ}}_{k}$: Sets of all subnetworks of size at most *k*; $R_{i},{\bar{R}}_{j}$: Subnetworks of the query networks; *N_k_*, *M_k_*: Numbers of all subnetworks of size at most *k*.

## Competing interests

The authors declare that they have no competing interests.

## Authors' contributions

FA, TK, and MD developed the method. MD and FA implemented the methods and gathered experimental results. FA and TK wrote the paper.
